# Determinants and effects of medical students’ core self-evaluation tendencies on clinical competence and workplace well-being in clerkship

**DOI:** 10.1371/journal.pone.0188651

**Published:** 2017-11-29

**Authors:** Yung Kai Lin, Der-Yuan Chen, Blossom Yen-Ju Lin

**Affiliations:** 1 Division of Cardiovascular Surgery, Cardiovascular Center, Taichung Veterans General Hospital, Taichung, Taiwan, ROC; 2 Department of Medical Education, Taichung Veterans General Hospital, Taichung, Taiwan, ROC; 3 Center of Rheumatology and Immunology, China Medical University Hospital, Taichung, Taiwan, ROC; 4 College of Medicine, China Medical University, Taichung, Taiwan, ROC; 5 School of Medicine, Medical Sociology, China Medical University, Taichung, Taiwan, ROC; Waseda University, JAPAN

## Abstract

Core self-evaluation (CSE) is a personality trait that involves a person’s evaluation of his or her own worth, competence, and capability. The objective of this study was to determine whether medical students’ CSEs exert beneficial effects on their adaptation to their clerkship in terms of their clinical competence and workplace well-being and whether their preclinical academic performance can be a trait-relevant situation that enhances their CSE expression. In total, 127 medical students from 2 cohorts were included as participants in this study. We analyzed complete measures of personal background, objective and subjective preclinical academic performance (course evaluation grades and self-reported efficacy), CSE tendencies, and clinical competence (as objective structured clinical examination scores) and workplace well-being (as compassion satisfaction and burnout) during their 2-year clerkship. Mixed linear models for repeated measures and multiple regressions were employed. Participants’ CSE tendencies had positive effects on their workplace compassion satisfaction and burnout but not on their clinical competence during their clerkship. Additionally, using the objective and subjective preclinical academic performance of the medical students as indicators, we observed that neither could be trait-relevant situations to enhance their CSE expression. CSE personality tendencies might be key to medical students’ ability to noncognitively adapt to clinical training during their clerkships. These tendencies should be identified earlier so that mentors can provide prompt care and support to mentees (medical students) during clerkships.

## Introduction

In recent years, core self-evaluation (CSE) has received attention in personality research. CSE is a personality trait that involves a person’s evaluation of his or her worth, competence, and capability [1]. CSE, which represents a broad concept of personality, describes an individual’s evaluation of themselves, their abilities, and their self-control, which covers 4 traits: self-esteem, general self-efficacy, locus of control, and emotional stability [2]. Principally in the field of organizational behavior, the effects of CSE on outcomes such as salary [3], goal setting [4, 5], job searching behavior [6], job satisfaction [6], job or task performance [4, 7–10], interpersonal relationships [11], career commitment [12], professional efficacy [13], and job burnout [12–16], have been verified in empirical studies. In the field of higher education, CSE has been verified to affect student behaviors, including test anxiety [17], academic performance [18, 19], academic burnout [20], entrepreneurial intentions [21], continued career commitment [22], and life satisfaction [23, 24].

Transitioning from preclinical education to clerkship entails challenges for medical students, one notable example is encountering real patients or scenarios instead of simulated patients or scenarios [25]. When experiencing the stress involved in transitioning from preclinical education to clinical training, medical students who perceive themselves as active actors in control of their future and who trust in their ability to influence their environment can improve their well-being [20, 26]. CSE involves cognitive or subjective appraisals and assumes that people hold viewpoints regarding themselves, other people, and the world, and it thus influences their personal appraisals of external events; therefore, CSE is particularly suited for occupational stress research [27]. Because of the broad application of CSE across educational and occupational fields, this personality trait might be a potential predictor of medical students’ adaptation from preclinical school to workplace clinical training during their clerkship. Therefore, the first hypothesis proposed in this study is as follows:

Hypothesis 1: Medical students’ CSEs affect their adaptation to their clerkship, including their clinical competence and workplace well-being.

Although CSE has been identified as a static personality trait that influences individuals’ work experiences, studies have determined that it can be influenced by trait-relevant situations [28–30]. For example, work experiences, such as satisfaction with the previous job, can predict CSE in later years [30] by encouraging employees to express their CSEs; thus, favorable work experiences help strengthen employees’ satisfaction levels in their job and lives [24]. Therefore, on the basis of trait activation theory [28, 29], we postulated our second hypothesis:

Hypothesis 2: Medical students’ preclinical academic performance is a situational clue that motivates their CSE expression to some extent.

## Materials and methods

Two research questions were addressed in this study: 1) whether medical students’ CSEs affect their adaptation to their clerkship in terms of their clinical competence and workplace well-being and 2) whether preclinical academic performance is a trait-relevant situation that enhances their CSE expression. Institutional review board approval was obtained for this prospective cohort study, and written informed consent was obtained from the participating medical students. Through online surveys, this study aimed to determine the clinical competence and well-being dynamics of medical students enrolled in a 2-year clerkship. For medical students who fulfilled our inclusion criteria on the basis of the online surveys, data on their preclinical academic performance were retrospectively collected. Ethical approval was granted by the China Medical University and China Medical University Hospital Ethics Committee [#CMUH102-REC3-088 and #CMUH102-REC3-088(CR-1)].

### Study participants and data collection

**Fig 1:**
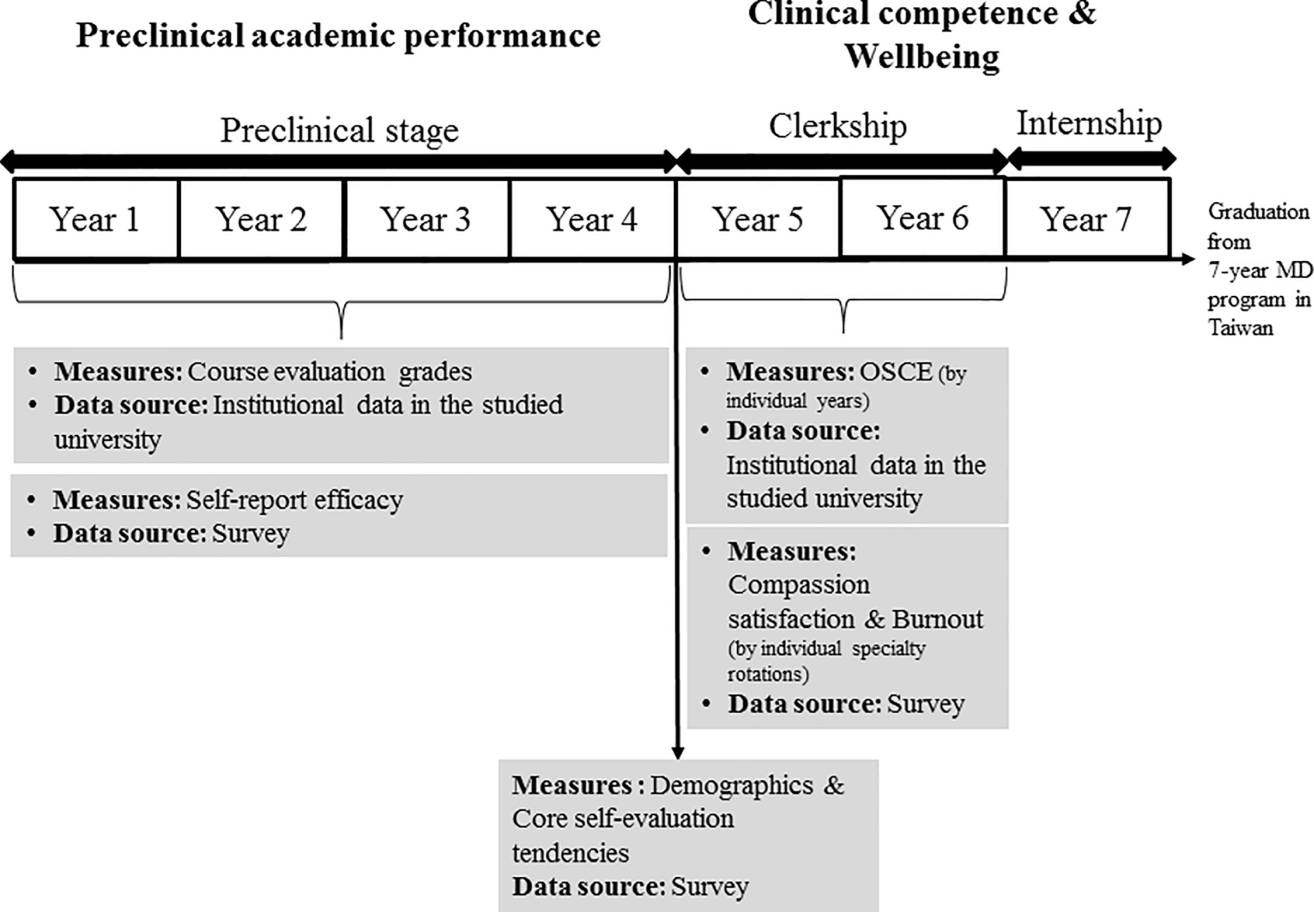
Timeline for data collection in this study.

The study participants were medical students from 2 cohorts who were studying in the 7-year doctor of medicine (MD) program of a university medical school in Taiwan and who had commenced their 2-year clinical workplace training clerkship in September 2013 and September 2014. All the training courses (in various specialties, which are listed in S1 Appendix) [31] of the 2-year clerkship were organized by the department of education of the university-affiliated medical center. Of a total of 258 medical students from the 2 cohorts, 154 students signed informed consent forms (participation rate: 60%). Subsequently, to obtain their demographic characteristics, perceived preclinical learning experience, and CSE properties, the participants were required to complete validated, structured, and self-administered questionnaires. After individual specialty rotations, routine questionnaires measuring compassion satisfaction and burnout, as indicators of medical students’ well-being, were distributed to prospectively collect the data during their 2-year clerkship. In addition, their objective structured clinical examination (OSCE) scores, as indicators of their clinical competence, were obtained prospectively and annually during their 2-year clerkship from the institutional data recorded in the university medical school. Because all participants were free to decide whether to complete the survey each time, participants who completed the surveys with at least 4 responses concerning compassion satisfaction and burnout with normality distributions across the clerkship were included in this study. Ultimately, 127 medical students were included in our study (participation rate: 49% [ie, 127 ÷ 258 × 100%]). The included participants provided 2,749 responses, ranging from 4 to 34 responses per person. The preclinical academic performance data of these 127 participants were retrospectively collected from the institutional data recorded in the university medical school. Fig 1 outlines the timeline for data collection.

### Study instrument

#### Preclinical academic performance of medical students (first- to fourth-year medical students)

Two types of preclinical academic performance data of the participants were used. First, course evaluation grades in the preclinical period provided by the faculty were used as the objective or expert scoring standard [32]. Courses in the medical school were divided into 7 categories: languages, general education, basic sciences, basic medicine, humanities and medicine, service learning, and preclinical organ-based medicine. Detailed course information for these 7 categories is listed in S2 Appendix. All the participants studied the courses (required and elective) throughout their preclinical education in the medical school for academic credits and were permitted to commence their workplace clinical training (ie, their clerkship) after they had passed all preclinical courses. Therefore, we used the final passing grades of medical students who retook their courses after failing them the first time to reflect their preclinical learning status and ability. The average of the grades for the aforementioned course categories was calculated for further analysis.

The trend toward competency-based medical education might lead students to learn not only from the formal curricula but also from other resources and individualized learning plans to meet their learning needs and assume responsibility for their learning [33, 34]. Therefore, course-teacher-rated grades might not completely reflect students’ abilities or their confidence in learning. Additionally, some confounding factors in their learning environment may interact to determine students’ confidence and achievements [35]. Therefore, in this study, the second type of preclinical academic performance data of students’ involved participants’ self-efficacy, which served as a subjective measure. It represented an alternative approach for participants to self-evaluate their performance (proficiency). Although some might argue against the accuracy of self-assessment, meta-analyses have revealed that students can moderately effectively self-assess their performance, can more accurately self-assess as they progress through medical school, and do not significantly overestimate or underestimate their own performance [32]. To describe their self-efficacy in preclinical academic performance, 4 items for classifying the major aspects of medical student learning, namely medical knowledge and judgment, medical technical skills, research training, and service learning [32, 36], were self-reported by the participants using a 5-point Likert scale (5 = *excellent*, 4 = *good*, 3 = *fair*, 2 = *poor*, and 1 = *very poor*).

#### Medical students’ CSE tendencies

CSE tendency was measured using the 12-item Core Self-Evaluations Scale [37] and a 5-point Likert scale (5 = *strongly agree*, 4 = *agree*, 3 = *neutral*, 2 = *disagree*, and 1 = *strongly disagree*). Confirmatory factor analysis (CFA) was performed for construct validity testing, and 2 items, for which the loadings were less than the 0.5, were excluded in this study, with a good fit at CMIN/DF = .242 (*P* > .05), GFI = 0.946, AGFI = 0.900, RMSEA = 0.044, PCLOSE = 0.557, HOELTER (0.05) = 148. Cronbach’s α value for the final 10 items was 0.824. The average of the 10 items was used for further analysis.

#### Medical students’ clinical competence in clerkship

Medical students’ clinical competence during their clerkship was measured using the OSCE scores obtained at the end of the first and second years of their clerkship. OSCE scores have been used as a reliable and suitable indicator of student performance, faculty teaching, and curriculum planning for achieving clinical competence [38–41].

#### Medical students’ workplace well-being in clerkship

Medical students’ workplace well-being refers to their compassion satisfaction and burnout as measured using the Professional Quality of Life (ProQOL): Compassion Satisfaction and Fatigue Version 5 scale [42]. ProQOL originally covered 2 major constructs, namely compassion satisfaction and compassion fatigue (which is further divided into 2 components, namely burnout and secondary traumatic stress) [42]. It has been increasingly employed in studies on physicians [43–45] and medical students [31, 46]. Most phenomena linked to secondary traumatic stress were unsuitable for the learning content and contexts of clinical clerkships. Therefore, in our study, we included only 2 constructs, namely compassion satisfaction and burnout. Compassion satisfaction refers to the positive feelings pertaining to a person’s ability to help others, whereas burnout is the negative effect of caring that is associated with feelings of hopelessness and difficulty in managing work or performing it effectively [47]. Both dimensions were measured using 10 items of the compassion satisfaction subscale, with rating on a 5-point Likert scale (5 = *always*, 4 = *often*, 3 = *sometimes*, 2 = *seldom*, and 1 = *never*). The construct validity of compassion satisfaction was determined through CFA, and all 10 items had a loading of more than 0.5 at statistical significance and were thus retained, with a good model fit at CMIN/DF = 6.639 (*P* < .05), GFI = 0.956, AGFI = 0.917, RMSEA = 0.045, PCLOSE = 0.893, HOELTER (0.05) = 608. Cronbach’s α value was 0.929 for the 10 items of the compassion satisfaction subscale. The construct validity of burnout was determined through CFA, and the 2 items, for which loadings were less than the .5, were excluded in this study, with a moderate model fit at CMIN/DF = 22.322 (*P* < .05), GFI = 0.936, AGFI = 0.856, RMSEA = 0.088, PCLOSE = 0.000, HOELTER (0.05) = 203. Cronbach’s α value was 0.780 for the final 8 items of the burnout subscale. The mean of the items for compassion satisfaction and burnout was subsequently used for further analysis.

#### Personal characteristics

Data on participants’ personal characteristics, including sex and age, were analyzed in this study.

### Data analysis

The data were analyzed by calculating various descriptive statistics, including the mean and standard deviation of continuous variables and the frequency and percentage of categorical variables.

The dependent variables (ie, OSCE scores, compassion satisfaction, and burnout) should be treated as correlated data because each participant was not an independent cohort and these repeated-measures data did not exhibit unequal variance and involved an unequal number of repetitions [48, 49]. Therefore, linear mixed-effects models were employed to determine whether medical students’ CSE influences their clinical competence and well-being during their clerkship (Hypothesis 1). Participants’ repeated OSCE scores, compassion satisfaction, and burnout were considered dependent, repeated variables; their CSE tendencies were considered independent variables; and their demographic characteristics (age and sex) were considered covariates.

Multiple regression analysis was performed to determine whether medical students’ preclinical academic performance in medical school might be a potential indicator of enhanced CSE (Hypothesis 2). In this analysis, individual medical students were units of analysis, and their CSEs were dependent variables; all the preclinical academic performance indictors were independent variables, and their demographic characteristics (age and sex) were covariates. Pearson correlation with r > 0.7 and VIF > 5 served as the cutoff points for potential multicollinearity of the independent explanatory variables [31, 50]. All analyses were performed using SPSS (IBM, Version 20.0).

## Results and discussion

Our study included 127 medical students from 2 cohorts. Women and men each comprised approximately half of the sample, and the average age was 23 years. The average of course evaluation grades (for 7 courses) was used to evaluate the preclinical academic performance of the participants, ranging from 84 to 90 (100 was the highest possible score). The self-reported efficacy of the participants regarding their preclinical academic performance in medical knowledge and judgment, medical technical skills, research training, and service learning ranged from 2.96 to 3.52 on the basis of the 5-point Likert scale. Participants’ measured CSE tendency was 3.18 on average on the basis of the 5-point Likert scale. In terms of participants’ clinical competence and well-being during the clerkship, the average OSCE scores in the first and second years of the clerkship were 376.46 and 406.68, respectively (600 was the highest possible score). The compassion satisfaction and burnout of the participants during the 2-year clerkship were 3.62 and 2.38, respectively, on the basis of the 5-point Likert scale. Table 1 presents descriptive analyses for all studied variables.

**Table 1:** Descriptive statistics of medical students’ demographics and core self-evaluation properties, preclinical academic performance, and clinical competence and well-being.

Variables	Scale	n	Mean	SD	Freq	%
***Demographics***						
Age		127	23.03	1.47		
Sex	Men	127			66	52
	Women				61	48
***Preclinical academic performance***						
**1. Course evaluation grades (objective indicators)**						
Languages	Score 0~100	127	86.19	5.59		
General education	Score 0~100	127	85.75	3.31		
Basic sciences	Score 0~100	127	84.79	4.09		
Basic medicine	Score 0~100	127	84.04	5.28		
Humanities and medicine	Score 0~100	127	86.00	3.30		
Service learning	Score 0~100	127	89.78	1.16		
Preclinical organ-based medicine	Score 0~100	127	84.01	6.30		
**2. Self-report efficacy (subjective indicators)**						
Medical knowledge and judgment	Score 1~5	127	3.24	0.72		
Medical skill	Score 1~5	127	2.96	0.93		
Research training	Score 1~5	127	3.04	0.87		
Service learning	Score 1~5	127	3.52	0.95		
***Core self-evaluation (CSE)***	Score 1~5	127	3.12	0.57		
***Clinical performance***						
OSCE (first-year clerkship)	Score 0~600	127	373.46	47.73		
OSCE (second-year clerkship)	Score 0~600	127	406.68	53.72		
***Clinical workplace well-beings***						
Compassion satisfaction (CS)	Score 1~5	2749	3.62	0.67		
Burnout (BO)	Score 1~5	2749	2.38	0.58		

### Effects of medical students’ CSE tendencies on their clinical competence and workplace well-being in clerkship

A linear mixed-effects model was employed to examine the effect of participants’ CSE tendencies on their clinical competence, as measured using OSCEs in the first and second years of their clerkship, as repeated measures (n = 127 × 2). The results revealed that participants’ CSE tendencies had no statistically significant effect on their clinical competence during the clerkship (*P* > .05).

Two linear mixed-effects models were separately employed to measure participants’ workplace well-being during their clerkship, as measured using compassion satisfaction and burnout, as repeated measures (n = 2,749). The results revealed that participants’ CSE tendencies had statistically significant and positive effects on their compassion satisfaction and burnout (*P* < .001). Additionally, older participants exhibited higher compassion satisfaction and lower burnout during their clerkship training than did their younger peers (*P* < .001). Men exhibited higher burnout and higher compassion satisfaction during clerkship training than did women (*P* < .001). Detailed information is provided in Table 2.

**Table 2:** Effects of medical students’ core self-evaluations on their clerkship competence and well-being: Linear mixed-models.

Parameters	Estimates	SE	P Value	95% confidence interval	
				lower	upper
**Repeated measures: OSCE scores in two-year clerkship (n = 254)**					
Core self-evaluation	5.441	5.893	0.357	-6.166	17.048
Age	-3.005	2.285	0.190	-7.507	1.496
Sex (default: women)	-7.445	6.768	0.272	-20.776	5.887
**Repeated measures: compassion satisfaction in two-year clerkship (n = 2,749)**					
Core self-evaluation	0.332	0.021	0.000	0.291	0.373
Age	0.043	0.008	0.000	0.028	0.059
Sex (default: women)	0.090	0.024	0.000	0.042	0.137
**Repeated measures: burnout in two-year clerkship (n = 2,749)**					
Core self-evaluation	-0.263	0.018	0.000	-0.299	-0.227
Age	-0.037	0.007	0.000	-0.050	-0.023
Sex (default: women)	0.107	0.021	0.000	0.066	0.149

### Determinants of medical students’ CSE tendencies: The role of preclinical academic performance in the medical school

To examine how participants’ preclinical academic performance is related to their CSE tendencies, course evaluation grades (7 items) and self-reported efficacy (4 items) were used as independent variables in a multiple regression model, with age and sex serving as covariates. Before regression modeling, the correlation between each of the independent variables (ie, preclinical academic performance) was examined for potential multicollinearity (Table 3). Finally, the course evaluation grades for basic science, exhibiting a high correlation with those for general education (γ = 0.741) and basic medicine (γ = 0.810), were excluded from the multiple regression model to avoid multicollinearity. The multiple regression analysis revealed that preclinical academic performance, objective or subjective, was unrelated to their higher CSE tendencies at the statistically significant level (*P* > .05). Detailed information is shown in Table 4.

**Table 3:** Correlation among all the measures of preclinical academic performance and demographics.

	(1)	(2)	(3)	(4)	(5)	(6)	(7)	(8)	(9)	(10)	(11)	(12)	(13)
**1. Course evaluation grades (objective indicators)**													
(1) Languages	1												
(2) General education	0.503***	1											
(3) Basic sciences	0.427***	0.741***	1										
(4) Basic medicine	0.282***	0.568***	0.810***	1									
(5) Humanities and medicine	0.455***	0.614***	0.635***	0.495***	1								
(6) Service learning	0.122	0.011	- 0.016	- 0.018	0.369***	1							
(7) Preclinical organ-based medicine	0.198*	0.446***	0.655***	0.783***	0.514***	0.070	1						
**2. Self-report efficacy (subjective indicators)**													
(8) Medical knowledge and judgment	- 0.003	0.151	0.241**	0.390***	0.092	0.026	0.356***	1					
(9) Medical skill	0.074	0.070	0.076	0.085	0.155	0.027	0.091	0.443***	1				
(10) Research training	- 0.032	0.094	0.117	0.166	0.104	0.122	0.197*	0.501***	0.488***	1			
(11) Service learning	0.023	0.056	0.006	0.047	0.103	0.042	0.085	0.276**	0.254**	0.408***	1		
**3. Demographics**													
(12) Age	- 0.044	0.004	0.036	- 0.049	0.072	0.011	- 0.026	- 0.127	0.018	- 0.135	- 0.086	1	
(13) Sex	0.162	0.058	0.041	0.027	0.098	- 0.052	- 0.006	- 0.168	- 0.044	- 0.094	0.101	- 0.096	1

*p<0.05

**p<0.01

***p<0.001

**Table 4:** Determinants of medical students’ core self-evaluation properties: Role of the preclinical academic performance in the medical school by multiple regression modeling.

Parameters	Std β	t Value	p Value
**Course evaluation grades (objective indicators)**			
Languages	0.039	0.359	0.720
General education	0.140	1.046	0.298
Basic medicine	-0.096	-0.585	0.560
Medical and humanities	-0.019	-0.133	0.894
Service learning	0.066	0.635	0.527
Preclinical organ-based medicine	-0.003	-0.020	0.984
**Self-report efficacy (subjective indicators)**			
Medical knowledge and judgment	0.064	0.527	0.599
Medical skills	0.102	0.915	0.362
Service learning	0.031	0.260	0.796
Research training	0.066	0.655	0.514
**Control variables**			
Age	-0.070	-0.759	0.449
Sex (men as default)	-0.166	-1.753	0.082

To avoid multicollinearity, one variable ─ the course evaluation grade of Basic science ─ was excluded.

### What we have learned from the findings?

As expected, this study revealed that participants’ CSE tendencies had positive effects on their workplace well-being—namely compassion satisfaction and burnout—during their clerkship. Previous studies have reported that positive CSE can mediate the relationship between self-insight and subjective well-being [51], the effect of dispositional optimism on life satisfaction of college students in China [23], the direct effects on the career commitment of medical school students [22] and higher professional efficacy and less burnout [13]. We postulate that CSE properties, which characterize personality traits including self-esteem, generalized self-efficacy, locus of control, and emotional stability, may be protective factors for the clinical training situations of medical students.

However, we did could not demonstrate that participants’ CSE enhanced their clinical competence performance during their clerkship. Although the OSCE scores of the participants varied, all participants passed the OSCE. The variation in OSCE scores may not have been sufficiently diverse to discriminate among the participants. Alternatively, participants’ clinical competence measured using the OSCE was cognitive and task-oriented and thus not relevant to soft, noncognitive personality tendencies, such as CSE tendencies measured in this study. Moreover, we could not determine the roles of neither preclinical academic performance in medical school or self-reported efficacy in preclinical academic performance. Additional studies should explore some of the potential trait triggers of CSE in medical students.

This study has several limitations. This study was limited to only one medical school; therefore, the study setting could be expanded in future studies to obtain generalizable results. Additionally, some might argue that learning can lead to personality changes that indicate psychological and professional maturation [52, 53] thus, medical students should be longitudinally traced from medical school admission to through the clinical training period. This cohort study design enables participants to be followed during their internship and postgraduate stages to assess their long-term medical career.

## Conclusions

In this study, we explored whether medical students’ CSE tendencies facilitate their adaptation to clerkship training and investigated the determinants of CSE tendencies. Our study results revealed that participants’ CSE facilitated their clinical adaptation to their clerkship in terms of compassion satisfaction and burnout but not clinical competence. Personality tendencies might be key to medical students’ noncognitive clinical training adaptation and should be identified earlier so that mentors can provide prompt care and support to their mentees (ie, the medical students) in clerkship training.

## Supporting information

S1 Appendix List of clinical specialties trained through the 2-year clerkship. (PDF)

S2 Appendix List of the preclinical curricula studied in the medical school. (PDF)

S1 Dataset Minimal anonymized data set. (XLSX)
