# Supplementary material for: Determinants and effects of medical students’ core self-evaluation tendencies on clinical competence and workplace well-being in clerkship
Source: PLoS One. 2017 Nov 29;12(11):e0188651. doi: 10.1371/journal.pone.0188651 (PMC5706685; doi:10.1371/journal.pone.0188651)
Supplement: S1 Appendix — (PDF) [file pone.0188651.s001.pdf]

Appendix 1. Lists of clinical specialties trained through 2-year clerkships [31].

The clinical specialties covered in the first-year clerkship were internal medicine (cardiology, chest medicine, hematology and oncology, infectious diseases, nephrology, gastroenterology, general medicine, and metabolism and endocrinology/rheumatology), psychiatry, neurology, surgery (general surgery, chest surgery, cardiovascular surgery, colon and rectal surgery, pediatric surgery, and trauma), obstetrics and gynecology, pediatrics, and radiology; those covered in the second-year clerkship were anesthesiology, orthopedics, neurosurgery, plastic surgery, urology, dermatology, rehabilitation medicine, ophthalmology, otolaryngology, family medicine, emergency medicine, and nuclear medicine/pathology/laboratory medicine/radiation oncology.
