# Supplementary material for: Determinants and effects of medical students’ core self-evaluation tendencies on clinical competence and workplace well-being in clerkship
Source: PLoS One. 2017 Nov 29;12(11):e0188651. doi: 10.1371/journal.pone.0188651 (PMC5706685; doi:10.1371/journal.pone.0188651)
Supplement: S2 Appendix — (PDF) [file pone.0188651.s002.pdf]

Appendix 2. List of the preclinical curricula studied in the medical school **(required and elective curriculum list in alphabetical order)**

Curriculum classification: Languages

Advanced English (reading, conversation, writing, and audiovisual)
Chinese (basic and advanced)
English (basic and advanced)
English Classics
English in Traditional Chinese Medicine
English Narratives
French (basic and advanced)
German (basic and advanced)
Japanese (basic and advanced)
Latin
Medical English Terminology
Practical English
Spanish (basic and advanced)

Curriculum classification: General education

Accounting
Administrative Science
Advertising
Aerobic Dance Training
Aesthetic Notes – Art Practice
Aesthetics of Movie Literature
American Culture
American Dance and Song
An Introduction to Archaeology
An Introduction to Political Science
Analysis and Musical Experience
Animal Behavior
Application of Nanobiotechnology
Application Software
Applied Aesthetics in Visual Art
Applied Chinese
Applied Ethics
Aromatherapy
Aromatic Horticulture
Aromatic Plants
Art and Humanistic Thinking
Arts and Culture
Arts and Life
Arts: Appreciation and Assessment
Behind the Nobel Prize
Belief and Life
Bioethics
Biographies of Figures in the Life Sciences
Biological Image
Buddha, Gilded Bronze
Buddhism
Business Law
Business Multimedia Design
Calligraphy

Care for People with Disability
Career Planning and Development
Career Planning and Survival Skills
Children and Society
Children's Literature and Readings
Chinese Art
Chinese Calligraphy
Chinese Classical Opera
Chinese Culture, Language, and Traditional Chinese Medicine
Chinese Herbal Medicine
Chinese Language in National Primary Schools
Chinese Myths and Legends
Chinese Philosophy
Chinese Seal Engraving
Cinema and Social Culture in Taiwan
City, Society, and Urban Space
Civil Law
Classic Reading
Classic Western Drama and Culture
Classic Western Literature
Classic Western Literature and Culture
Classical Medical Reading - Formula Poems in Traditional Chinese Medicine
Classical Medical Reading-Nan-Ching
Classical Music
Classical Poetry
Cognitive Psychology
Composition of Digital Music
Computer Networks
Computer Networks (Introduction)
Computer-Aided Melody Creation
Computing
Constitution of the R.O.C.
Constitutional Law
Consultation (Introduction)
Contemporary Basic Global Environmental Issues
Contemporary Science and Social Change
Contemporary Social Problems and Analyses
Cosmetics
Craft Art and Practice
Crisis of Parasites
Cultural Anthropology
Cultural Studies
Culture and Art in Taiwanese History
Culture and Life
Database Design
Democracy and Law
Democratic Politics and Modern Society
Development of Chinese Herbs
Developmental Psychology
Dialogues between the Mind and the Brain
Digital Informatics
Disaster

Disease and Civilization: Infectious Disease, Human History, and Science
Diversity of Plants
Drama
Drawing
Drawing and Practice
East Asian Civilization: Tradition and Modernity
E-commerce
Economics
Education, Media, and Technology
Electronic Data Processing
English Composition
English for Environmental Science
English for Medicine and Nursing
English Prose
English Reading
English Writing
Environment and Disaster Prevention
Environment and Disease
Essence of the Historical Records: a Selective Reading
Ethical Issues in the Life Sciences
Ethics and Emerging Technologies
Experiments in Manipulating and Differentiating Human Embryonic Stem Cells
Expressive Art and Emotional Management
Famous Western Novels
Female Screenwriters and Writers
Fiction and Novels
Film and Philosophy
Film Production
Financial Management of Risk
Flower Essences
Food Service Management
Formal Letter Writing
Formal Letter Writing
Formative Design
Formosa Treasure Island: Places and Stories
Formosan Medicine
Fractals
Free Radicals and Diseases
Frontier Biotechnology
Frozen Food
Gender and Society
Gender Relationships
Gender Relationships in Chinese Literature
General History of China
General Principles of Law
General Psychology
General Sociology
Geography, Migration, and Development
Global Art Museums
Global Citizens: A Selected Literature Review
Global Environmental Change and Sustainable Development
Global Gender Issues

Global Health
Global Issues: An Ecological Perspective
Global Society and Civic Education
Greek and Roman Mythology
Green Environment Mindset
Health and Safety (Introduction)
Health Care and Civilization
Health Care Cost Analysis
Health Care Economics
Health Care Law
Health Care Nutrition
Health Care with Meridian Theory
Health Food Marketing
Health Laws and Regulations
Health Policy and Analysis
Health Policy and Hospital Management: Taiwan vs. the United States
Health Promotion and Disease Prevention
Health Psychology
Health, Illness, and Medicine
Heritage: History in Taiwan
Historical Mindset
Historical Mindset and Seminar on Chinese History
Historical Mystery Analysis
Historical Simulations and Reconstruction
Historical Thinking and Seminar on Taiwanese History
History
History and Thought
History of China
History of Chinese Pharmacy
History of Chinese Philosophy
History of European Cultures
History of Humankind: from Human Evolution to Genome Project
History of Medical Education in Taiwan
History of Natural Taiwan
History of Taiwan
Human Evolution
Human Health Risk Management
Human Resources and Social Development
Human Resources Management
Humanistic Physical Therapy
Image, Visual, and History
Immune Science Technology
Industrial Hygiene
Industrial Management
Industrial Safety
Information Literacy and Interdisciplinary Studies
Integrated Human Resources in Long-Term Care
International Business English Communication
International Relationships
Internet Marketing
Interpersonal Relationships
Investment Science

Japanese for Oral Health Science
Journalistic English: Reading and Analyzing
Jurisprudence
Language and Thought
Language Art
Law and Life
Learning for Connoisseurs and Collectors of Art
Life and Bioinformatics
Life and Ethics
Life and Existence
Life Art
Life Science Seminar
Life, Human Dignity, and Law
Life: A Priceless Treasure
Literary
Literature and Cinema
Literature of Theater
Literature Review
Living English for Medicine
Logic
Long-term Care
Long-term Care Management
Management
Mandarin Speaking (basic and advanced)
Marketing Management
Marketing Management for Health Care Organizations
Marriage and Family
Media, Democracy, and Global Communication
Medical Classics
Medical English
Medical English and Writing
Medical Law and Regulations
Medicines
Methods of Medical Research
Modern Chinese Fiction
Modern Essay Writing
Modern Novels
Modern Poetry
Modernity and Postmodernism
Movies
Movies and Literature
Multicultural Education
Multimedia and Hypermedia
Music
Music and Arts
Music and Culture
Music and Human Life
Music and Literature
Music and Movies
Music Theory
Music, Language, and the Brain
Musical Composition

Musical Cultures of the World
Mysteries of Flowers and Fruits
Mysteries of History
Mysterious Insect World and Human Beings
Nano-biology
Nano-chemistry
Nantechnology
Nature Conservation
Neuroscience
Nobel Prize in Physiology and Medicine
Nongovernmental Organizations
Notable Figures in History
Novels
Nursing Administration
Nutrition and Cooking
Nutrition and Health
Nutrition and Pregnancy
Nutrition for Long-term Care Residents
Office Automation Software
Official Document Writing
Opera
Oral Health Industry
Organic Foods
Parasites and Life
Pedagogy
Pentsaology
Pharmaceutical Marketing
Pharmacy in Real Life
Pharmacy Management
Philosophy
Philosophy and Contemporary Thought
Philosophy of Zhuang-gi
Plant Ecology
Poise and Grace
Poisonous Plants
Political and Economic Development in Taiwan
Politics, Law, and Modern Society Lectures
Population, Geography, and Society
Principles and Techniques of Photography
Principles of Bodily Care
Principles of Economics
Probiotics and Health Care
Professional Conduct for Medical Doctors
Programming Languages
Progress and Predicaments of Germany in Modern and Contemporary History
Prose
Prosocial Behavior Theory and Practice
Psychology in Film
Public Health
Quality Management in Health Care
Reading and Thinking of Historical Documents
Reading and Writing

Reading and Writing: Medicine -related Literature
Reading and Writing: Poetry
Reading Classic Children's Literature
Reading English Newspapers
Readings of Selected Six Dynasties' Fiction
Rehabilitation and Exercise in Long-term Care
Reproduction of Plants
Research on Social Change and Tendencies
Rhetoric and Oral Presentation Skills
Risk Assessment and Management
Science, Information, and Life
Scientific and Technical English
Seal Carving Art
Security Education and First Aid
Selected Readings from Western Essayists
Selected Readings in Taiwanese Literature
Selected Readings of Short Stories
Seminar in Masterpieces of Art
Seminar on Humanitarian Assistance
Shakespeare
Shakespeare in the Modern Time
Shakespeare–Classic and Modern
Social Culture and Health Care
Social Movements: their Roles and Prospects in Taiwanese History
Social, Ethical, and Legal Implications in Biomedicine
Society, Culture, and Health Care
Sociology
Sociology of the Family
Special Lecture on Pharmacy
Special Topic in Measurement: Item Response Theory
Steps in Writing
Study of Knight-Errant Novel Culture
Study of Life and Death
Studying Abroad
Survey and Society
Swordsman Fiction
Taiwan's Foreign Relations: from 1949 to the present
Taiwanese Cinema and Modern Thought
Taiwanese Herbal Medicine
Taiwanese Medical History
Taiwanese Modern Drama
Taiwanese Movies and Literature
Taiwanese Music
Taiwanese Traditional Theatrical Arts (An Introduction)
Tax Law
Technology and Human Beings
Technology, Humanity, and Society
The Civil Culture of Taiwanese History
The Three Kingdoms: History and Fiction
Theater
Theater and Life
Theory and Practice of Counseling

Theory and Research of Intellectual Property
Theory and Technique of Counseling
Toxic Plant Taxonomy
Traditional Chinese Medicine and Culture
Traditional Chinese Medicine Classical Literature
Traditional Chinese Medicine in Life
Traditional Herbal Diet
Visual Art
Water Pollution Control
Welfare Services for Senior Citizens and People with Disability
Western Drama
Western Literature
Western Literature and Love
Western Modern Art
Western Music
Western Philosophy
Western Short Stories
WHO and International Public Health Law
Women's Studies
Working with Families
World Studies
WTO and Globalization
Yijing Feng Shui and Landscape

Curriculum classification: Basic sciences

Accident Prevention and Control
Actuarial Science
Acupuncture
Acupuncture Nursing
Analytical Chemistry
Analytical Chemistry Laboratory
Anticancer Drug Discovery
Antimicrobial Agents
Application of Animal Model of Vascular Reocclusion in Chinese Herbal Medicine
Application of Computers in Biomedical Science
Applied Mathematics
Artificial Biology
Assisted Reproduction Technology
Beauty and Nutrition
Bioinformatics
Biological Evolution and Systematics
Biomaterials
Bio-mathematics
Biomechanics
Biomedical Engineering
Biomedical Signal Imaging
Biophysics
Biosignal Measurement
Biostatistics
Biostatistics Practice
Biotechnology
Biotechnology of Food Science
C programming and Image Processing

Calculus
Cellular and Molecular Regulation and Molecular Evolution Analysis
Chinese Crude Drugs
Community Health
Computed Tomography: Principles and Applications
Computer- Aided Drug Design
Cosmetic Materials Science
Cosmetic Psychology
Cosmetology
Demography and Health Statistics
Developmental Biology
Diet Planning and Management
Dietary Fiber
Drug Delivery
Drug Design in Signal Pathway
Drug Information
Drug Management
Drug Synthesis
Ecology
Engineering Mathematics
Environmental and Occupational Epidemiology
Environmental Chemistry
Environmental Chemistry Laboratory
Environmental Health
Environmental Microbiology
Environmental Toxicology
Enzymology
Epidemiology
Ethnobotany
Evolutionary Developmental Biology
Experiments on Biological Activity of Traditional Chinese Herbal Medicine
Experiments on Inducible Pluripotent Stem Cell Generation
Financial Management
Fluid Mechanics
Folk Medicine in Taiwan
Food Additives
Food and Health
Food Hygiene and Safety
Food Processing
Food Science Technology
Frontiers in Biomedicine
Functional Foods
General Biology
General Biology Laboratory
General Chemistry
General Chemistry Laboratory
General Physics
General Physics Laboratory
Health and Environmental Laws
Health Care System
Health Food
Health Insurance and Payment System

Health Policy and Politics
Health Services Management
Health Services Management Seminar
Health Sociology
Healthy Food
Herb Dietetics
Herb Pharmacology
Herb Processing
Herb Processing Laboratory
Herbal Pharmacy of Traditional Chinese Medicine
Histopathologic Techniques
Hospital Administration
Hospital Management Seminar
Human Development
Human Evolution
Identification of Chinese Drugs
Independent Research
Instrumental Analysis
Java Language Programming
Laboratory Animals
Laboratory Toxicology
Leading-edge Science and Cutting-edge Technology
Life Nutrition
Macromolecular Structural Analysis by Computer
Management of Athletic Training
Medical Administration and Management
Medical Engineering
Medical Mathematics
Medical Physics
Medical Record Management
Medical Statistics
Medical Technology
Medical Terminology
Medicine and Health
Modern Diet Therapy
Natural Health
Non-prescription Drugs
Nutrition
Nutrition Administration
Nutrition and Beauty
Nutrition and Diet Therapy
Nutrition and Disease
Nutrition and Free Radical
Nutrition and Immunity
Nutrition in Traditional Chinese Medicine
Nutritional Supplements
Organic Chemistry
Organic Chemistry Laboratory
Organizational Behavior
Origin of Human Diseases
PERL Programming and Bioinformatics Applications
Pharmaceutical Botany

Pharmaceutical Botany Laboratory
Pharmaceutical Mathematics
Pharmaceutical Mineralogy
Pharmaceutical Zoology
Pharmacology
Pharmacology Laboratory
Pharmacology Laboratory in Traditional Chinese Medicine Pharmacy
Pharmacy Orientation
Physical Chemistry
Physics of Medical Imaging
Practical Chinese Herbology
Practice in Human Development
Principles of Foods
Principles of Foods Laboratory
Processing of Chinese Crude Drugs
Proteomics
Proteomics Laboratory
Public Health
Public Health Administration
Public Health Response to Emergency Events
Quality Control of Traditional Chinese Medicine
Research Methodology
Research Methodology of Biotechnology
School Health
Scientific Writing
Signal Transduction
Social Research Methods
Special Topics of Traditional Chinese Medical and Nutritional Therapy
Special Topics on Acupuncture Studies: Electrophysiological Aspects
Special Topics on Perception and Attention
Sports and Leisure
Sports Nutrition
Sports Psychology
Study on Biological Activity of Traditional Chinese Herbal Medicine
Systems Biology
Thermodynamics
Tissue Culture
Tissue Engineering
Toxicology
Toxicology of Traditional Chinese Medicine
Traditional Chinese Medical Pharmacology
Traditional Chinese Medicated diet
Traditional Chinese Medicinal Materials
Traditional Chinese Medicine Experiment
Traditional Chinese Medicine Prescription
Traditional Chinese Pharmacy
Traditional Chinese Prescriptions and Material Medicine
Ultrasound
Vector Control
Vegetarian Nutrition

Curriculum classification: Basic medicine

Hospice Care

Immunology
Industrial Microbiology
Infectious Diseases
International Classification of Disease
Laboratory Genetics
Literature of Traditional Chinese Medicine
Maternal and Child Health
Medical Genetics
Medical Image Processing
Medicine
Metabolism and Diseases
Microbial Genetics
Microbiology and Immunology
Microbiology and Immunology Laboratory
Molecular Biology
Molecular Medicine
Nei-Ching (Classical Literature of Traditional Chinese Medicine)
Neuroanatomy
Neuroanatomy Laboratory
Neuroanatomy Practice
Nutrition and Aging
Nutrition and Cancer
Occupational and Environmental Medicine
Oncology Patient Care
Oral Health of Special Needs Patients
Oral Microbiology
Oral Oncology
Oral Pathology
Pain Science
Parasitology
Parasitology Laboratory
Pathology
Pathology Laboratory
Pathophysiology
Pediatrics of Traditional Chinese Medicine
Pharmacology
Pharmacology Laboratory
Pharmacology: Applied Physiology
Pharmacology: Drug Action and Therapy
Physical Assessment
Physical Medicine and Rehabilitation
Physical Therapy
Physiology
Physiology Laboratory
Pilates Training
Prescriptions and Herbal Pharmacy of Traditional Chinese Medicine
Prevention of Occupational Disease
Psychiatry
Psychiatry and Mental Health
Pulse Sphygmology
Qi-gong
Radiation Biology

Regenerative Medicine
Rehabilitation and Nutrition
Special Topical on Physiological Research
Sports Massage
Sports Tui-na Science
Strength Training
Symptoms and Treatments in Traditional Chinese Medicine
Theory of Chin-kuei (Miscellaneous Diseases)
Theory of Shang-Han (Chinese Pyretology)
Traditional Chinese Medicine
Traditional Chinese Traumatology in Nursing
Translational Medicine
Traumatology of Traditional Chinese Medicine
Tumor Biology
Wen-Bing (Classical Epidemic Febric Diseases)

Curriculum classification: Humanities and medicine

Being a Friend to Patients
Bioethics
Biomedical Ethics
Clinical Communication Skills
Clinical Ethics
Communication Theory and Skills
Comprehension and Compassion of Life
Dialogue between Humanities and Medicine
English Conversation for Medicine
Health Administration
History of Chinese and Western Medicine
History of Medicine
History of Traditional Chinese Medicine
Industrial Psychology
Life and Ethics
Medical Ethics
Medical Malpractice: Theory and Practice
Medical Movies
Medical Practice Law
Medical Problem Solving and Analysis
Medical Professions
Medical Psychology
Medical Reasoning and Critical Thinking
Medical Seminar
Medicine and Sociology
Multi-reflection upon Ethical Issues
Physician and Society
Practice of Community Medicine
Psychology
Social Psychology
Standing by the Patient
Taiwanese Language for Medicine
The Meaning of Life and Death

Curriculum classification: Service learning

Service learning

Curriculum classification: Organ-based integrated medicine

Advanced Cardiac Life Support
Anatomy in Medical Imaging
Anesthesiology
Cardiology
Cardiovascular Surgery
Chest Medicine
Chest Surgery
Chinese Internal Medicine
Clinical Cases in Emergency Medicine
Clinical Diagnosis and Pre-clerkship
Clinical Diagnostic Methods
Clinical Diagnostics
Clinical Neurophysiology
Clinical Pathological Seminar
Clinical Simulation
Clinical Special Radiology
Cosmetic Dermatology
Dermatology
Electrocardiogram
Emergency Medicine
Endocrinology and Metabolism
Family Medicine
Gastroenterology
General Surgery
Gerontology
Hematology
Image Diagnosis
Infectious Diseases
Internal Medicine
Laboratory Medicine
Nephrology
Neurology
Neurosurgery
Nuclear Medicine
Obstetrics and Gynecology
Ophthalmology
Osteology
Otorhinolaryngology
Palliative Care
Pediatric Surgery
Pediatrics
Plastic Surgery
Radiation Oncology
Radiation Oncology
Radiation Technology
Radiology
Rehabilitation Medicine
Rheumatology Immunology Allergy
Surgery
Surgical Oncology
Urology
